# Fatigue characteristics on dialysis and non-dialysis days in patients with chronic kidney failure on maintenance hemodialysis

**DOI:** 10.1186/s12882-021-02314-0

**Published:** 2021-03-27

**Authors:** Subrata Debnath, Rain Rueda, Shweta Bansal, Balakuntalam S. Kasinath, Kumar Sharma, Carlos Lorenzo

**Affiliations:** 1 Division of Nephrology, Department of Medicine, University of Texas Health San Antonio, 7703 Floyd Curl Dr, San Antonio, TX USA; 2 University Health, 4502 Medical Dr, San Antonio, TX USA; 3 Division of Clinical Immunology, Department of Medicine, University of Texas Health San Antonio, 7703 Floyd Curl Dr, San Antonio, TX USA

**Keywords:** Fatigue, Severity, Interference, Dialysis day, Non-dialysis day

## Abstract

**Background:**

Fatigue is prevalent in hemodialysis patients who for survival follow a strict dialysis treatment regimen – dialysis and non-dialysis days. As a result, the daily activities, symptom burden, and clinical outcomes of hemodialysis patients vary significantly between dialysis and non-dialysis days. Fatigue is one of the most reported debilitating symptoms by hemodialysis patients with profound negative impact on their quality of life. Prior studies assessed fatigue during the preceding 7 or 30 days and did not discriminate fatigue characteristics between dialysis and non-dialysis days. We aimed to characterize and compare fatigue severity and fatigue interference with daily activities between dialysis and non-dialysis days.

**Methods:**

Hemodialysis patients self-reported fatigue on consecutive dialysis and non-dialysis days using the 9-item Brief Fatigue Inventory. The differences in fatigue characteristics between dialysis and non-dialysis days were analyzed using one-way ANCOVA.

**Results:**

Global fatigue burden was worse on a dialysis day compared to a non-dialysis day (*P* for all < 0.001). Age and education were associated with fatigue, but hemodialysis-related variables were not. A significant inverse association of physical activity with fatigue severity observed on non-dialysis day; there was also a negative association between the normalized protein catabolic rate and fatigue severity on both dialysis and non-dialysis days. The positive association of depression with fatigue severity and fatigue interference were consistent on both dialysis and non-dialysis days. None of these factors, however, explained differences in fatigue characteristics between dialysis and non-dialysis days.

**Conclusions:**

Fatigue, measured in severity and interference, was more pronounced on a dialysis day relative to a non-dialysis day. These differences were not explained by age, sex, education, hemodialysis-related variables, habitual exercise, nutritional status, and or depression. The quantitative measures of fatigue characteristics may facilitate future interventional trials design and better fatigue management for hemodialysis patients.

## Background

Kidney failure patients on maintenance hemodialysis treatment endure multitude of symptoms and rank fatigue as one of the most dreadful symptoms which adversely affects their daily activities and quality of life [1–4]. These patients prioritize relief of fatigue over survival [2, 5]. For survival, hemodialysis patients follow a strict weekly treatment regimen – either Monday-Wednesday-Friday or Tuesday-Thursday-Saturday are dialysis days (treatment days) and remaining days are non-dialysis days (non-treatment days). Due to such unique and restrictive life pattern, the daily activities, symptom burden, and clinical outcomes of hemodialysis patients vary significantly between dialysis and non-dialysis days. For example, hemodialysis patients consume remarkably lower dietary energy and protein, experience diminished mood and impaired cognitive function, and report worse subjective well-being on dialysis days compared to non-dialysis days [6–8]. Studies also showed higher relative risks for mortality on dialysis days compared to non-dialysis days [9]. However, little is known about the fatigue characteristics and magnitude of fatigue burden on dialysis and non-dialysis days.

Prior studies measured fatigue over the preceding week or month [10]. Diurnal fatigue course and pattern over days have also been reported [11, 12]. However, these studies did not discriminate the characteristics of fatigue between dialysis and non-dialysis days. Fatigue severity and the pervasive impact of fatigue on life participation are the two most important dimensions of fatigue [10, 13]. The differences in fatigue severity and fatigue interference with daily activities, mood, relations with family and friends, life enjoyment, etc. between dialysis and non-dialysis days have not been well characterized. The magnitude of global fatigue burden on dialysis and non-dialysis days is also unknown.

Contemporary reports emphasize such knowledge gap and critically advocate for more explicit research geared toward a better understanding of fatigue [14–16]. Elucidating the difference of fatigue on both dialysis and non-dialysis days may help design future research studies and clinical trials to implement targeted and timely intervention to mitigate fatigue burden. Therefore, the purpose of this study was to characterize and compare day-to-day fatigue severity and fatigue interference with daily activities in hemodialysis patients.

## Methods

### Study design and population

This observational study enrolled clinically stable prevalent hemodialysis patients treated at two in-center dialysis clinics with following eligibility criteria: (i) on thrice weekly hemodialysis dialysis for at least six months; and (ii) without clinical or laboratory diagnosis of malnutrition and anemia, acute cardiovascular events requiring hospitalization, and comorbidities such as active malignant cancer, refractory psychiatric disorders, and significant neurological disorders. All patients received standard management for hemodialysis and comorbidities as per the recommended guidelines. Each patient was dialyzed for an average of 4 h with high-flux polysulfone dialyzers using bicarbonate based dialysate. The study was approved by the local Institutional Review Board and all participants provided written informed consent prior to the study procedures.

### Measurements

#### General measurements

Patients’ medical charts were reviewed to obtain routine dialysis day blood chemistry values and dialysis parameters including dialysis adequacy measure, i.e., Kt/V. Body weight and blood pressure values were collected on a dialysis day at two time points – at the beginning and end of the dialysis session. To minimize variabilities, all study procedures were performed during the mid-week hemodialysis treatment.

A self-administered questionnaire was utilized to collect socio-demographic data on ethnicity, educational attainment, and employment status. In addition, self-reported weekly time spent on physical activities was recorded. To assess depression, a 21-item Beck Depression Inventory (BDI)-II was administered on a dialysis day [17]. The BDI-II assesses symptoms of depression during the past two weeks. Each BDI-II item represents a symptom with a scale value of 0 (no symptom) to 3 (severe symptom) and summing the total scores range from 0 to 63 – higher scores represent more severe depression [17]. BDI-II is an extensively validated and widely used depression screening instrument in hemodialysis patients [18].

#### Brief Fatigue Inventory

The Brief Fatigue Inventory (BFI) [19] was used to document self-reported fatigue. During a mid-week dialysis session, each participant received two sets of BFI – one marked with “Dialysis Day” and the other with “Non-dialysis Day” for treatment and non-treatment day, respectively. Each participant completed the “Dialysis Day” BFI within the first hour of dialysis session. The “Non-dialysis Day” BFI was completed on the following day within the same timeframe – approximately 24 h later. Depending on the day (dialysis or non-dialysis), fatigue reporting time for all nine BFI items was fixed to the specific day [19]. BFI was not administered to capture fatigue immediately after dialysis session. The first three BFI items measure fatigue severity level on a 0–10 scale with score ranging from 0 (no fatigue) to 10 (fatigue as bad as you can imagine). The BFI item #3 “fatigue worst” score can be utilized to classify ‘severe fatigue’ (score of ≥7) and ‘non-severe fatigue’ (score of < 7) for conceptual simplicity [19]. The remaining six BFI items assess fatigue interference in relation to patients’ general activity, mood, walking ability, normal work (both indoor and outdoor), relations with other people, and enjoyment of life on a 0–10 numerical rating scale, with 0 being “does not interfere” and 10 being “completely interferes.” The arithmetic means of the first three and last six BFI items were used to define fatigue severity and fatigue interference score, respectively. The arithmetic mean of the nine BFI items was used as a global fatigue score. BFI has been extensively validated in cancer patients [19] and used in hemodialysis patients [20].

### Statistical analysis

Fatigue scores on a dialysis day were compared to those on a non-dialysis day using one-way ANCOVA in order to account for the effect of relevant socio-demographic, laboratory, and dialysis parameters. We also assessed the influence of dietary protein on fatigue, estimated by the normalized protein catabolic rate (nPCR), which is often used as a measure of habitual dietary protein intake and normalized to the patient’s body weight [21, 22]. We used following formula to calculate nPCR [23]:

$$ \mathrm{nPCR},\mathrm{in}\ \mathrm{g}/\mathrm{kg}\ \mathrm{per}\ \mathrm{day}=0.22+\frac{\left(0.036\times \mathrm{in}\mathrm{tradialytic}\ \mathrm{rise}\ \mathrm{in}\ \mathrm{blood}\ \mathrm{urea}\ \mathrm{nitrogen}\times 24\right)}{\mathrm{intradialytic}\ \mathrm{in}\mathrm{terval}\ \left(\mathrm{hours}\right)} $$

Pearson correlation coefficient was used to examine the strength of the relationship of fatigue scores between dialysis and non-dialysis days, and the relationship of socio-demographic, exercise, nPCR, laboratory variables, dialysis parameters, and BDI-II scores with fatigue scores on both dialysis and non-dialysis days.

Variables that were associated with fatigue were used as covariates in analysis that examined fatigue differences between dialysis and non-dialysis days. We used one-way ANCOVA to assess differences in fatigue severity, fatigue interference, and global fatigue between dialysis and non-dialysis days in order to account for the effect of variables that were associated with fatigue. Among the covariates, age, exercise, nPCR, and BDI-II score were used as continuous variables. All analyses were performed using the SAS (version 9.4, SAS Institute Inc., Cary, NC). A two-sided *P* value < 0.05 was considered statistically significant.

## Results

### Population characteristics

Of total 209 maintenance hemodialysis patients from two in-center dialysis centers, 127 met the study eligibility criteria and 115 provided written consent to participate in the study. The characteristics of the study participants are shown in Table 1. The mean age was 54.8 ± 12.8 years and 47.8% were female. The mean serum albumin, nPCR, and blood hemoglobin levels of the study participants were 3.46 ± 0.38 g/dl, 1.03 ± 0.30 g/kg/day, and 11.04 ± 1.43 g/dl, respectively. The primary etiology of kidney failure for all subjects was type 2 diabetes and the mean hemoglobin A1c of the study patients was 6.93 ± 1.86%.

**Table 1:** Characteristics of the study participants, *n* = 115

Variable	
Age, yr	54.8 ± 12.8
Female, %	47.8
Education (%)	
**≤** 8th grade	33.3
9th grade to **≤**high school	19.3
High school diploma	32.5
Vocational school or some college	11.4
Bachelor degree	2.6
Graduate degree	0.9
Exercise (min/week)	59.8 ± 87.6
Pre-dialysis body weight, kg	81.64 ± 20.66
Pre-dialysis body mass index, kg/m^2^	31.07 ± 7.70
Pre-dialysis systolic blood pressure, mmHg	151.11 ± 26.94
Pre-dialysis diastolic blood pressure, mmHg	71.43 ± 15.40
Dialysis adequacy (Kt/V)	1.84 ± 0.38
Duration of dialysis, months	56.76 ± 39.60
Normalized protein catabolic rate, g/kg per day	1.03 ± 0.30
Blood urea nitrogen, mg/dL	53.09 ± 14.41
Serum albumin, g/dL	3.46 ± 0.38
Phosphorus, mg/dL	5.33 ± 1.61
Hemoglobin A1c, %	6.93 ± 1.86
Hemoglobin, gm/dL	11.04 ± 1.43
Hematocrit, %	34.55 ± 4.22
Intact parathyroid hormone, pg/mL	332.24 ± 324.05
Vitamin D, pg/mL	45.17 ± 25.74

^*^Data are mean ± standard deviation, or *n* (%)

### Characteristics of fatigue on dialysis and non-dialysis days

**Fig. 1:**
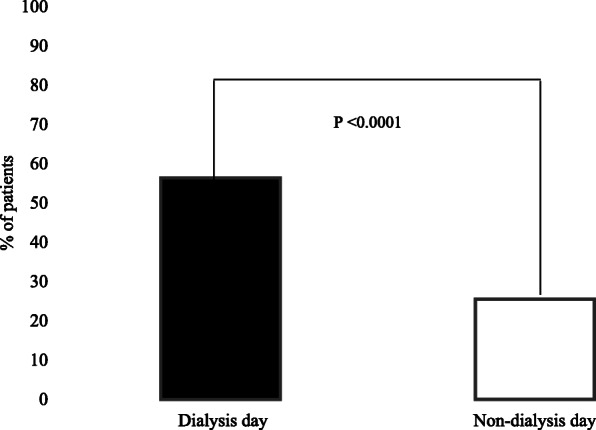
Prevalence of fatigue severity on dialysis and non-dialysis days (Fatigue severtiy defined as Brief Fatigue Inventory item #3 “worst” score of > 7)

Fatigue severity and fatigue interreference scores on both dialysis and non-dialysis days are presented in Table 2. The unadjusted mean scores for fatigue now, usual, and worst were all remarkably higher on a dialysis day compared to a non-dialysis day. Fatigue severity level (mean of first 3 BFI items scores) was significantly pronounced on a dialysis day compared to a non-dialysis day, 5.35 ± 2.50 and 3.47 ± 2.85, *P* < 0.0001, respectively (Table 2). Prevalence of severe fatigue (defined as BFI item #3 “fatigue worst” score ≥ 7) is displayed in Fig. 1.

**Table 2:** Fatigue characteristics on dialysis and non-dialysis days

Fatigue characteristics	Dialysis Day	Non-dialysis Day	P^*^	P^**^	P^***^
*Severity*					
Fatigue (weariness, tiredness) NOW	4.39 ± 2.75	3.23 ± 2.86	0.003	0.001	0.002
USUAL level of fatigue (weariness, tiredness)	5.21 ± 3.03	3.23 ± 2.86	< 0.0001	< 0.0001	< 0.0001
WORST level of fatigue (weariness, tiredness)	6.36 ± 2.75	3.96 ± 3.11	< 0.0001	< 0.0001	< 0.0001
*Interference: fatigue interfered with*					
General activity	5.14 ± 3.05	2.88 ± 2.87	< 0.0001	< 0.0001	< 0.0001
Mood	4.65 ± 3.07	2.97 ± 3.09	< 0.0001	< 0.0001	< 0.0001
Walking ability	4.67 ± 3.27	3.53 ± 3.35	0.01	0.01	0.025
Normal work (includes both work outside the home and daily chores)	4.79 ± 3.09	3.35 ± 3.24	0.001	0.0006	0.0011
Relations with other people	3.83 ± 3.39	2.75 ± 3.26	0.02	0.01	0.024
Enjoyment of life	4.68 ± 3.41	3.23 ± 3.43	0.002	0.001	0.001
*Mean score*					
Fatigue level (severity)	5.35 ± 2.50	3.47 ± 2.85	< 0.0001	< 0.0001	< 0.0001
Fatigue interference	4.55 ± 2.65	3.14 ± 2.92	0.0005	0.0002	0.0002
Global fatigue	4.82 ± 2.43	3.25 ± 2.80	< 0.0001	< 0.0001	< 0.0001

Data are mean ± standard deviation

^*^Unadjusted

^**^Adjusted for age, sex, education, dialysis day, dialysis shift (morning, afternoon, or evening), and duration on hemodialysis

^***^Adjusted also for exercise, depression, and nPCR

**Fig. 2:**
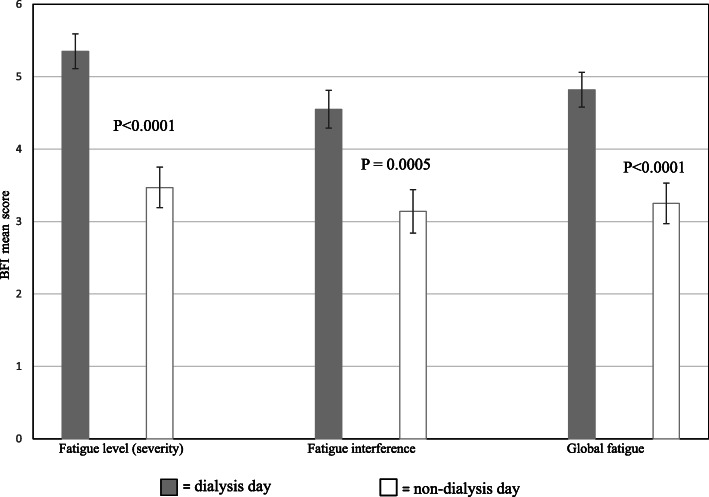
Mean (+ SE) scores of fatigue severity, fatigue interference, and global fatigue on dialysis and non-dialysis days

Similar trend was noted for fatigue interference items with life participations (Table 2). Among these, impact of fatigue on general activity and mood were strikingly higher on a dialysis day compared to a non-dialysis day, *P* for both < 0.0001. Fatigue severity (mean score of last 6 BFI items) on hemodialysis patients’ daily life activities was more severe (*P =* 0.0002) on a dialysis day relative to a non-dialysis day. Dialysis and non-dialysis fatigue severity, fatigue interference, and global fatigue burden are presented in Fig. 2.

### Univariate analysis for correlates of fatigue characteristics on dialysis and non-dialysis days

Correlates of fatigue were examined in order to identify variables that could explain differences in fatigue between dialysis and non-dialysis days. The associations of fatigue with age, years of education, habitual physical activity or exercise, nPCR, and BDI-II scores are presented in Table 3. There was no consistent pattern of correlations observed except for BDI-II which was positively and significantly associated with fatigue severity, fatigue interference, and global fatigue burden on both dialysis and non-dialysis days.

**Table 3:** Pearson correlation coefficients relating fatigue characteristics to age, education, exercise, BDI-II, and nPCR on dialysis and non-dialysis days

Fatigue characteristics		Age	Education	Exercise	BDI-II	nPCR
Severity	Dialysis day	0.21^*^	– 0.19	– 0.21	0.30*	−0.25^*^
	Non-dialysis day	0.14	– 0.25^*^	– 0.36^*^	0.43^**^	−0.24^*^
Interference	Dialysis day	0.21^*^	– 0.22^*^	– 0.20	0.41^**^	−0.18
	Non-dialysis day	0.28^*^	– 0.22^*^	– 0.25	0.45^**^	−0.12
Global fatigue	Dialysis day	0.23^*^	– 0.21^*^	– 0.22	0.40^**^	−0.23^*^
	Non-dialysis day	0.24^*^	– 0.24^*^	– 0.29^*^	0.47^**^	−0.16

*BDI-II* Beck Depression Inventory-II, *nPCR* normalized protein catabolic rate

^*^ *P* < 0.05

^**^P < 0.0001

We noted a significant inverse association of physical activity with fatigue severity and global fatigue on non-dialysis day only. There was a trend towards negative association between physical activity and fatigue interference on non-dialysis day (r = − 0.25, *P* = 0.08). nPCR negatively correlated with fatigue severity (both dialysis and non-dialysis days) and global fatigue burden (on dialysis day only) (Table 3). In contrast, the significant positive association of depression with fatigue severity, interference, and global fatigue burden was consistently found on both dialysis and non-dialysis days.

Pre- and post-dialysis body weight and blood pressure as well as intradialytic changes in body weight and blood pressure did not correlate with dialysis day fatigue characteristics. Likewise, dialysis vintage, dialysis adequacy or Kt/V, and routinely measured laboratory parameters including serum albumin, blood hemoglobin, and vitamin D levels did not correlate either with dialysis day fatigue characteristics including global fatigue burden (*P* for all > 0.05).

### Effects of factors on fatigue differences between dialysis and non-dialysis days

Differences in significance in fatigue severity, fatigue interference, and global fatigue between dialysis and non-dialysis days did not change after the adjustment for the following potential confounding factors: age, sex, education, dialysis shift (morning, afternoon, or evening), and duration of hemodialysis dialysis. Adjustment for additional variables such as dietary protein intake as measured by nPCR, time spent on habitual physical activity, and depression symptoms did not alter the robustness in differences in fatigue scores between dialysis and non-dialysis days (Table 2).

## Discussion

Fatigue severity and fatigue interference with daily activities are the two most important fatigue characteristics reported by the hemodialysis patients [13]. Our study results quantitively demonstrate that both fatigue severity and fatigue interference with daily activities are significantly greater on a dialysis day than on a non-dialysis day. Study data illustrate a high prevalence of worst fatigue on a dialysis day. The burden of global fatigue is overwhelming on a dialysis day relative to a non-dialysis day. Our analysis suggests that the factors which are associated with fatigue do not explain differences in fatigue between dialysis and non-dialysis days (Table 2).

The prevalence of fatigue has been extensively studied. However, fatigue characteristics, its determinants, and differences in fatigue characteristics between dialysis and non-dialysis days are poorly understood. Prior studies examined the ‘diurnal’ fatigue pattern without characterizing fatigue severity and fatigue interference on dialysis and non-dialysis days. For example, one qualitative study [12] described fatigue as ‘never-ending’ over a 36-h period meaning fatigue persists on non-dialysis day. Abdel-Kader et al. [11] documented significantly higher ‘fatigue-exhaustion-feeling sleepy’ symptom score on a dialysis day compared to a non-dialysis day in 55 patients using the ecological momentary assessment (EMA) method for consecutive 7 days. Recently, Brys et al. [24] employed similar EMA method to study ‘fatigue course’ in 51 hemodialysis patients and found that on a dialysis day fatigue increased significantly compared to a non-dialysis day; however, these studies did not examine the fatigue characteristics. Moreover, the EMA method, while appealing in certain conditions, requires significant time to complete – a major disadvantage for participants who are fatigued (e.g., hemodialysis and cancer patients) [25] and with low health literacy such as patients in our study [26]. Nevertheless, our findings of global fatigue burden on both dialysis and non-dialysis days are consistent with these prior studies.

The reason for pronounced fatigue severity and fatigue interference and overwhelming global fatigue burden on a dialysis day relative to a non-dialysis day has not been well investigated. One can postulate a number of factors (sociodemographic, lifestyle, physiological, and psychological) to explain such day-to-day fatigue variations. We noted a positive association of fatigue with age and inverse association with education which were not consistent across dialysis and non-dialysis days (Table 3). In accordance with prior studies, [15, 20, 27, 28] we did not find any association of select biochemical parameters (serum albumin, blood hemoglobin, and vitamin D) or any dialysis-related variables (changes in body weight and blood pressure) on a dialysis day with fatigue (e.g., severity and interference).

The negative association between habitual exercise or physical activity and fatigue only on non-dialysis days is not a surprising finding. Prior studies reported that hemodialysis patients are significantly less engaged in physical activities on a dialysis day compared to a non-dialysis day due to several reasons – notably, lack of motivation, time commitment during dialysis days including travel to dialysis clinic, and post-dialysis fatigue [29–31]. In addition, it is likely that chronic kidney failure patients with primary etiology of diabetes suffer from multiple comorbidities such as diabetic retinopathy, peripheral neuropathy, lower limb amputation, and arthropathy which are significant barrier to physical activity [32]. Self-reported mean duration of time spent on habitual physical activity by our study participants was only about 60 min per week.

It is also possible that significantly lower dietary energy and protein intakes on dialysis days compared to non-dialysis days may contribute to fatigue [6]. Burrowes et al. [6] showed that both dietary energy and protein intakes were lower on dialysis day than on non-dialysis day in the Hemodialysis study patients. We found that nPCR, which is often used to evaluate habitual protein intake, [33, 34] was inversely associated with global fatigue on dialysis day and with fatigue severity on both dialysis and non-dialysis days (Table 3). These findings are consistent with results from a recent study [35]. In general, a low-protein diet may contribute to malnutrition and skeletal muscle loss resulting in poor outcomes including fatigue. The mean nPCR of our study population was 1.03 ± 0.30 g/kg/day (Table 1) which was below the optimal target of > 1.4 g/kg per day and the Kidney Disease Outcomes Quality Initiative clinical practice guidelines recommend minimum target of 1.2 g/kg/day [33, 34].

We found that depression was the only factor that correlated significantly with fatigue severity and interference on both dialysis and non-dialysis days. However, there are no comparative data available to corroborate our findings. Several studies reported significant association between fatigue and depression with moderate effect size and the nature of this relationship is yet to be elucidated [14, 36]. There are at least two caveats to our findings. First, discrimination of fatigue and depression from each other is difficult using the currently available survey instruments because of overlapping psychological symptoms [14]. In fact, the BDI-II contains two fatigue items – loss of energy (item #15) and tiredness (item #20). Second, BDI-II was administered only once – on dialysis day. It could be speculated that hemodialysis patients may also experience diurnal or day-to-day variation in depression symptoms as has been reported in other patient population [37], which was not examined in the present study. Nevertheless, our observations warrant further exploration of patterns of depression using time sensitive tool on both dialysis and non-dialysis days, which could help understand the association of fatigue and depression.

It should be emphasized that the aforementioned factors (age, education, depression, exercise, and nPCR) were associated with fatigue characteristics (Table 3), but did not explain the differences in fatigue characteristics between dialysis and non-dialysis days. As can be seen from the Table 2, the unadjusted robust differences in fatigue scores between dialysis and non-dialysis days remained unchanged after adjusting for potential confounders. To our knowledge there is no comparative study available to validate our findings.

Our study has some strengths. One strength is the use of BFI. Unlike other fatigue measures, the BFI used in this study captures fatigue during the past 24 h or present day which is appropriate for the assessment of day-to-day fatigue variability unique to hemodialysis patients. In addition, BFI appears to meet the expressed recommendations of the Standardized Outcomes in Nephrology-Hemodialysis consensus workshop [13] for meaningful measurement of fatigue [38]. Of interest, the 3-question SONG-HD Fatigue Instrument [39], published recently, captures fatigue during the past week, missing unique day-to-day fatigue variations. It should also be noted that SONG-HD Fatigue Instrument has not been tested in US hemodialysis patients – the outcomes and mortality of whom are significantly different than that of Australia or United Kingdom [40]. Another strength is that our study patients were homogenous with regard to the primary etiology of kidney failure. The lack of heterogeneity of our sample population may have reduced the impact of particular disease-related factors on the results [41]. There are some limitations to this study as well. First, the sample size of the study was relatively modest. Second, homogenous patient population may limit the generalizability of the study findings. Third, we acknowledge that the correlations between fatigue and some modifiable variables (e.g., habitual exercise and protein intake) were modest and not consistent on both dialysis and non-dialysis days. Since we did not assess daily differences in exercise and protein intake, we cannot state that these are not important in explaining fatigue differences between dialysis and non-dialysis days. Prior studies reported significant variations in exercise and nutritional status on dialysis days compared to non-dialysis days [6, 31]. Fourth, we did not collect any laboratory or clinical parameters during the non-dialysis day which precludes precise association with and comparison of fatigue characteristics between dialysis and non-dialysis days. Future studies should measure these variables on both days in a larger and diverse patient sample to improve generalizability, and understand better which factors contribute to fatigue on dialysis and non-dialysis days. Inclusion of ultrafiltration, diffusion, osmotic shifts, and other intradialytic hemodynamics often implicated in post-dialysis fatigue were not investigated in the current study. They may help explain fatigue differences between dialysis and non-dialysis days.

Despite limitations, our findings may have clinical implications. Daily adequate amount of quality protein intake along with regular physical activity may alleviate fatigue burden especially on dialysis days and should be promoted by lifestyle or behavioral modifications. As mentioned, hemodialysis patients are less engaged in physical activities on dialysis days and preliminary results demonstrate that low-to-moderate-intensity exercise prior to hemodialysis is effective in improving fatigue [42]. One small-scale randomized clinical trial also demonstrated intradialytic exercise was efficacious in reducing fatigue in the intervention group compared to the control group [43]. From clinical perspectives, our results reinforce the importance of maintaining the recommend minimum target of nPCR of 1.2 g/kg/day. Intradialytic protein intake improves nPCR [44] and it may be worthwhile to examine the clinical efficacy of improved nPCR in alleviating fatigue. Of note, adequate protein intake, expressed as nPCR, has an independent salutary effect on morbidity and mortality in hemodialysis patients despite maintaining adequate dialysis dose [45]. It is unknown if clinical management of depression would be efficacious to relieve fatigue severity. It has been shown, however, that anti-depressant medication leads to a significant improvement in nPCR level and is effective in treating depression in hemodialysis patients [46]. Our findings may improve the understanding of fatigue toward the development of reliable fatigue measurement scale for clinical use. These results may also pave the way for the development of guidelines to define and manage fatigue in hemodialysis patients.

## Conclusions

This study extends our current knowledge of the nature of fatigue by demonstrating its characteristics on both dialysis and non-dialysis days. Our results show that fatigue severity and the negative impact of fatigue on daily activities – general activity, mood, walking ability, normal work, relations with other people, and enjoyment of life – were remarkably pronounced on a dialysis day compared to a non-dialysis day. In addition, severe fatigue was highly prevalent on a dialysis day. The study shows significant relationships between fatigue and physical activity, nutritional status, and depression, but these correlates do not explain differences in fatigue between dialysis and non-dialysis days in the patient cohort we studied and further research is needed. These findings may help both clinical trialists and researchers design experiments and trials with targeted and timely interventions leading to improved fatigue management in hemodialysis patients.

## Data Availability

The datasets generated and/or analyzed during the current study are not publicly available because the study is not federally funded and study activities are ongoing and data are being analyzed; however, data may be available from the corresponding author on reasonable request.
